# Nanodiscs Allow Phage Display Selection for Ligands to Non-Linear Epitopes on Membrane Proteins

**DOI:** 10.1371/journal.pone.0072272

**Published:** 2013-09-09

**Authors:** Marina Pavlidou, Karen Hänel, Luis Möckel, Dieter Willbold

**Affiliations:** 1 Institute of Complex Systems (ICS-6), Forschungszentrum Jülich, Jülich, Germany; 2 Institut für Physikalische Biologie, Heinrich-Heine-Universität Düsseldorf, Düsseldorf, Germany; Centro Nacional de Biotecnologia - CSIC, Spain

## Abstract

In this work, we exploited a method that uses polytopic membrane proteins as targets for phage display selections. Membrane proteins represent the largest class of drug targets and drug discovery is mostly based on the identification of ligands binding to target molecules. The screening of a phage display library for ligands against membrane proteins is typically hindered by the requirement of these proteins for a membrane environment, which is necessary to retain correct folding and epitope formation. Especially in proteins with multiple transmembrane domains, epitopes often are non-linear and consist of a combination of loops between transmembrane stretches of the proteins. Here, we have used bacteriorhodopsin (bR) as a model of polytopic membrane protein, assembled into nanoscale phospholipid bilayers, so called nanodiscs, to screen a phage display library for potential ligands. Nanodiscs provide a native-like environment to membrane proteins and thus selection of ligands can take place in a near physiological state. Screening a 12-mer phage display peptide library against bR nanodiscs led to the isolation of phage clones binding specifically to bR. We were further able to identify the binding site of selected phage clones proving that the clones bind to extramembranous, non-linear epitopes of bR. Thus, nanodiscs provide a suitable and general tool that allows screening of a phage display library against membrane proteins in a near native environment.

## Introduction

Membrane proteins are of particular biological importance, because they play essential roles in the cells of all living organisms. They are for example involved in the transport of substances across the membrane, in cell signaling, energy transduction, metabolic pathways and much more. Genome analysis of numerous organisms revealed that 20–30% of all open reading frames are predicted to encode for membrane proteins [1]. Membrane proteins are of special interest in the pharmaceutical and biotechnological industry, indeed 60% of all clinical drugs target membrane proteins [2]. Identifying ligands that modulate the activity of membrane proteins is of great interest for the discovery of novel drugs and diagnostic agents.

Phage display peptide libraries are an effective tool for the selection of peptide ligands binding a target protein [3]. Randomized peptides are displayed on the surface of filamentous phages by fusing random oligonucleotide sequences in frame with the coding sequence of the phage coat protein pIII or pVIII creating a library of more than 10^9^ different clones. The selection of clones out of this library that bind the target is traditionally achieved by “panning” the library against the purified, immobilized target. Modifications of the classical protocol have been made in several works to select for ligands with particular characteristics. By competitive selection strategies it is possible to select ligands that are highly specific for a given protein, but not for closely related proteins [4]. Mirror image phage display is a modification of the “common” phage display approach that allows the selection of ligands consisting solely of d-amino acid residues [5]–[7]. Such so called d-peptides are considered to be more suitable for diagnostic and therapeutic approaches, because they are more resistant to proteolytic degradation.

But despite the wide range of modifications, which can be applied on this method, the use of membrane proteins in phage display selections remains a challenge. Nevertheless, there are examples where membrane proteins have been used as targets in phage display selections. These were, however, very special and non-generic examples and were restricted to certain membrane proteins. It was shown that the detergent solubilized envelope glycoprotein of HIV-1, a type-1 integral membrane protein, can be used for the selection of antibodies from a phage display library [8]. However, for membrane proteins with more than one transmembrane domain the solubilization with detergents is problematic because of reasons described below. Other approaches have been developed for this purpose. For example, whole cells that stably express the membrane protein of interest can be used as a panning agent [9] allowing the target protein to exist in its native conformation. Unspecific binding of phages to other membrane proteins on the cell surface might be a problem of this technique. Also, whole-cell panning favors the selection of ligands against extracellular regions of the receptor, whereas selection of intracellular binding peptides requires internalization of the phages [10]. Other possibilities for phage display selection targeting membrane proteins are in vivo selection [11] or the screening of soluble fragments of the membrane protein [12]. Especially for G-protein coupled receptors (GPCR), however, the integration of the whole protein in the membrane is crucial for the formation of binding sites, so that soluble extracellular fragments of the receptor are not necessarily suitable as targets.

Full-length recombinant membrane proteins might be more suitable as targets in phage display selections. Their preparation, however, is problematic. Because of their need for a membrane environment and their strong hydrophobicity, membrane proteins require the reconstitution into membrane model systems to prevent aggregation. Membrane model systems are for example detergent micelles, liposomes, bicelles and nanodiscs. Detergents usually provide the primary solubilization step in the preparation of membrane proteins. They solubilize the membrane protein by forming micelles around the protein and because of their amphipathic character they mimic to some extent the natural bilayer of the membrane. The use of a given detergent to reconstitute a membrane protein, however, bears the risk of destabilizing the protein [13] and of affecting substrate binding [14]. The correct alignment of non-linear epitopes formed by polytopic membrane proteins can also be affected in detergent micelles. Membrane models consisting of lipids resemble the natural membrane more closely than detergents. A widely used membrane mimicking model are liposomes. Here, the protein is kept in a membrane environment by incorporation into spherical lipid bilayer vesicles. Liposomes, however, are prone to aggregation, instability and size heterogeneity. Bicelles are model membranes that are composed of a central planar lipid bilayer formed by long-chain phospholipids, while detergents or short-chain phospholipids surround the rim protecting the hydrophobic parts of the long-chain phospholipids from the aqueous solution. Bicelles, however, show heterogeneity concerning shape, size and composition [15].

In our studies, we have used nanodiscs as a model membrane to reconstitute a polytopic membrane protein and further screen a phage display peptide library for peptides binding to the membrane protein of choice. Nanodiscs are nanometer-sized phospholipid bilayers encircled by two copies of an amphipathic α-helical protein, called membrane scaffold protein (MSP) [16], [17]. Incorporated into nanodiscs the membrane proteins are more stable than in liposomes and bicelles and soluble in a detergent-free environment [18]. Structural and functional techniques can thus be applied to membrane proteins, which previously were limited to soluble proteins [19]–[21]. The nanodisc size distribution is reported to be homogeneous and most often monodisperse, in contrast to other model membranes [18]. Unlike in liposomes, membrane proteins in nanodiscs are accessible from both sides of the membrane. Membrane proteins in nanodiscs are located in a native-like environment and retain correct folding. Immobilization of membrane proteins on a solid support [22], which is necessary for phage display selection, is easily feasible with nanodiscs.

We have used bacteriorhodopsin (bR) from the archaebacterium *Halobacterium salinarum* as target protein for the phage display selection. We chose bacteriorhodopsin as a model system for membrane proteins with multiple transmembrane domains. Just like GPCRs, which form the largest family of drug targets, bR possesses seven transmembrane helices connected by small loops. bR is also well characterized concerning its spectroscopic and biophysical properties. In previous works by Bayburt and his co-workers it was shown that bR can be assembled into nanodiscs in monomeric and oligomeric forms [23], [24].

## Materials and Methods

### Materials

1,2-dimyristoyl-*sn*-glycero-3-phosphocholine (DMPC) was obtained from Avanti Polar Lipids (Alabaster, AL, USA). Size exclusion chromatography (SEC) was conducted on an ÄKTApurifier (GE Healthcare, Freiburg, Germany).

### Preparation of nanodiscs

The preparation of empty nanodiscs was carried out as described elsewhere [16]. Briefly, the membrane scaffold protein MSP1, expressed and purified as published previously [19], was mixed as 80–120 µM solution with a 50 mM solution of DMPC solubilized in 20 mM Tris-HCl pH 7.4, 100 mM sodium cholate in a molar ratio of 1∶70. The mixture was then incubated alternately at 4°C and 25°C (20 min each) followed by dialysis over 16 hours against 3×0.5 l of 20 mM Tris-HCl pH 7.4, 100 mM NaCl, 0.5 mM EDTA using a membrane with a molecular weight cut off of 1000 Da. After dialysis, the mixture was loaded onto a HiLoad XK16/600 Superdex 200 column (GE Healthcare, Freiburg, Germany) equilibrated with 10 mM sodium phosphate pH 7.4, 150 mM NaCl as running buffer and a flow rate of 1 ml/min. Fractions containing nanodiscs were pooled and concentrated up to 200–250 µM using Centriprep centrifugal filter units (Merck, Millipore, Darmstadt, Germany) and Vivaspin 2 centrifugal concentrators (Sartorius, Göttingen, Germany) both with a molecular weight cut off of 10 kDa. The concentration of the nanodiscs was determined by UV absorbance at 280 nm using an extinction coefficient corresponding to two molecules of MSP1 (ε = 2×23950 = 47900). The stability of the nanodiscs was confirmed by analytical SEC on a Superdex 200 10/300 GL column (GE Healthcare, Uppsala, Sweden) with a flow rate of 0.6 ml/min and 10 mM sodium phosphate pH 7.4, 150 mM NaCl as running buffer.

### Cell-free expression of bR nanodiscs

For cell-free expression of bacteriorhodopsin (bR), the expression plasmid pEXP5-CT/bR (Invitrogen, Carlsbad, CA, USA) was used. The stop codon (TAG) of the bR encoding gene was mutated into TCG using the Quick Change II Site-Directed Mutagenesis Kit (Stratagene, La Jolla, CA, USA) allowing expression of bacteriorhodopsin with C-terminal His_6_-tag. Cell-free protein synthesis reactions were carried out using Expressway Maxi Cell-Free *E. coli* Expression System (Invitrogen, Carlsbad, CA, USA) following the manufacturer's instruction. The reactions were up-scaled to a volume of 1 ml. Additionally, 5 µM all trans-retinal and 1.35 mg/ml nanodiscs were added to each reaction [25]. Expression was carried out for 2.5 h in total at 37°C and 1200 rpm.

### Purification of bR nanodiscs

After expression, the cell-free reaction was centrifuged for 10 min at 15000× g to obtain the soluble fraction. bR containing nanodiscs were purified from the soluble fraction by nickel-nitrilotriacetic acid (Ni-NTA) agarose (QIAGEN, Hilden, Germany). Therefore, the soluble fraction was incubated with the resin for 30 min at room temperature following by washing with increasing concentrations of imidazole from 0–20 mM in 50 mM sodium phosphate pH 8, 500 mM NaCl. Bound protein was eluted from the column with 250 mM imidazole in the same buffer. All fractions were analyzed by SDS-PAGE. Elution fractions containing bR nanodiscs were used for preparative SEC on a HiLoad XK16/600 Superdex 200 column (GE Healthcare, Uppsala, Sweden) with a flow rate of 0.5 ml/min and 10 mM sodium phosphate pH 7.4, 150 mM NaCl as running buffer. The column was calibrated with five standard proteins (thyroglobulin, ferritin, aldolase, conalbumin, ribonuclease A) of known molecular weight and, except for conalbumin, of known Stokes diameter. Void volume was determined with blue dextran. The elution volumes were used to generate a calibration graph allowing the determination of molecular weight and Stokes diameter. Total protein content was monitored at a wavelength of 280 nm, whereas the presence of bacteriorhodopsin was monitored at 550 nm. Fractions containing bR loaded nanodiscs were used for further experiments and were checked for stability and homogeneity on a Superdex 200 10/300 GL column (GE Healthcare, Uppsala, Sweden) with a flow rate of 0.6 ml/min. Absorption spectra of bR loaded nanodiscs were collected in a Lambda 25 UV/Vis spectrophotometer (Perkin Elmer, Boston, USA).

### Immobilization of nanodiscs

Nunc Immobilizer Amino 96 well micro titer plates (Thermo Fisher Scientific, Waltham, MA, USA) were coated over night at 8°C with 100 µl of loaded or unloaded nanodiscs (10 µg/ml diluted in phosphate buffered saline (PBS)). After washing with PBS+0.05% Tween 20, the plate was treated with 10 mM ethanolamine in 100 mM sodium carbonate pH 9.6 for 1 h at room temperature followed again by washing with PBS+0.05% Tween 20. Remaining binding sites were blocked with 1% BSA (bovine serum albumin) in tris buffered saline (TBS)+0.1% Tween 20 for 1 h at room temperature. The plates were washed six times with TBS+0.1% Tween 20 before used for panning or antiphage ELISA.

### Panning of the 12-mer random peptide display library

A commercially available phage display library based on random peptide 12-mers fused to the minor coat protein (pIII) of M13 phage was used to perform the biopanning (Ph.D.™-12 Phage Display Peptide Library, New England Biolabs, Inc., Ipswich, MA, USA). For each selection round, 2×10^11^ phages diluted in TBS+0.1% Tween 20 were added to a well of Nunc Immobilizer Amino plate, that was previously coated with bR loaded nanodiscs, and incubated for 20 min at room temperature. After 10× washing with TBS+0.1% Tween 20, bound phages were eluted by incubating the well for 10 min with 0.2 M Glycine-HCl pH 2.2. Eluted phages were neutralized with 1 M Tris-HCl pH 9.1. Phage titering and amplification of the phages were carried out according to manufacturer's instructions (Instruction manual, version 2.7).

### Determination of bR binding of single affinity-purified phage clones (antiphage ELISA)/Sequencing

bR loaded nanodiscs and unloaded nanodiscs were immobilized on Nunc Immobilizer Amino 96 well micro titer plates as described above. To test binding of phages to BSA-blocked empty wells, 100 µl PBS were added to the wells, incubated overnight at 8°C and further treated like the wells that were incubated with target. Binding of single phage clones was analyzed by adding 100 µl of amplified phage clones as culture supernatant to the wells and incubated them for 1.5 h at room temperature. Each single phage clone was added to wells coated with PBS, loaded and unloaded nanodiscs, respectively. After washing six times with TBS+0.1% Tween 20, 200 µl of a horseradish peroxidase-conjugated anti-M13 monoclonal antibody (GE Healthcare, Uppsala, Sweden) solution (1∶5000 dilution in TBS, 0.1% Tween 20, 1% BSA) were added and incubated for 1 h. Plates were washed again six times with TBS+0.1% Tween 20 and binding of the phages was detected via the horseradish peroxidase conjugated to the anti-M13 antibody using 3,3′,5,5′-tetramethylbenzidine (Sigma Aldrich, St. Louis, MO, USA) as a substrate.

Single stranded phage DNA was purified as described in the NEB phage display manual (Instruction manual, version 2.7) with the exception that the iodide buffer was exchanged by a 10∶1 mixture of 3 M sodium acetate pH 5.2 and TE buffer. Sequencing was done by seqlab, Göttingen, Germany.

### Identification of binding sites

Peptides representing the loop regions and termini of bacteriorhodopsin were purchased from JPT Peptide Technologies GmbH (Berlin, Germany) (table 1). All peptides, except of the N-terminus, were amino-terminally biotinylated. The N-terminus was carboxy-terminally biotinylated and amino-terminally acetylated. A PEG-based spacer arm (N-(3-{2-[2-(3-Amino-propoxy)-ethoxy]-ethoxy}-propyl)-succinamic acid) was attached between the peptide and the biotin to minimize steric hindrance for binding on streptavidin coated plates. All peptides were carboxy-terminally amidated. In addition to their linear forms, peptides that represent loops of bacteriorhodopsin were also synthesized as cyclic forms by flanking them with cysteines. Although the linear peptides can adopt any conformation, the respective cyclic form may increase binding due to a more favorable entropic term of the binding energy. Because the cyclic form may be more closely to the native loop conformation in bR, we decided to use cyclic forms of the loop peptides as well.

**Table 1:** List of peptides resembling the extramembranous regions of bR.

name	Sequence
**N-terminus**	Acetyl-QAQITGRPEW-Ttds-Lys(Biotin)NH_2_
**loop AB**	Biotin-Ttds-KGMGVSDPDAKKFY-NH_2_
**loop AB cyclic**	Biotin-Ttds-CKGMGVSDPDAKKFYC-NH_2_
**loop BC**	Biotin-Ttsd-GYGLTMVPFGGEQNPI-NH_2_
**loop BC cyclic**	Biotin-Ttsd-CGYGLTMVPFGGEQNPIC-NH_2_
**loop CD**	Biotin-Ttds-LALLVDADQGT-NH_2_
**loop CD cyclic**	Biotin-Ttds-CLALLVDADQGTC-NH_2_
**loop DE**	Biotin-Ttds-TKVYSYR-NH_2_
**loop DE cyclic**	Biotin-Ttds-CTKVYSYRC-NH_2_
**loop EF**	Biotin-Ttds-GFTSKAESMRPEVASTFK-NH_2_
**loop EF cyclic**	Biotin-Ttds-CGFTSKAESMRPEVASTFKC-NH_2_
**loop FG**	Biotin-Ttds-GSEGAGIVPLNI-NH_2_
**loop FG cyclic**	Biotin-Ttds-CGSEGAGIVPLNIC-NH_2_
**C-terminus**	Biotin-Ttds-LRSRAIFGEAEAPEPSAGDGAAATS-NH_2_

Peptides were biotinylated to allow capturing on streptavidin coated plates. Ttds ((N-(3-{2-[2-(3-Amino-propoxy)-ethoxy]-ethoxy}-propyl)-succinamic acid) was used as spacer between the biotin and the peptide to reduce steric hindrance. All peptides were C-terminally amidated.

56 pmol of peptides were coated over night at 8°C on streptavidin coated plates (Thermo Fisher Scientific, Waltham, MA, USA). After blocking with 1% BSA in TBS+0.1% Tween 20 for 1 h, the plates were washed with PBS+0.05% Tween 20 and incubated for 1.5 h with 100 µl of phages from bacterial supernatant. After 6 washing steps with PBS+0.05% Tween 20, the anti-M13 antibody solution (1∶5000 dilution in TBS, 0.1% Tween 20, 1% BSA) was added and binding was detected as it was done for the anti-phage ELISA.

## Results

### Assembly of bacteriorhodopsin into nanodiscs

**Figure 1:**
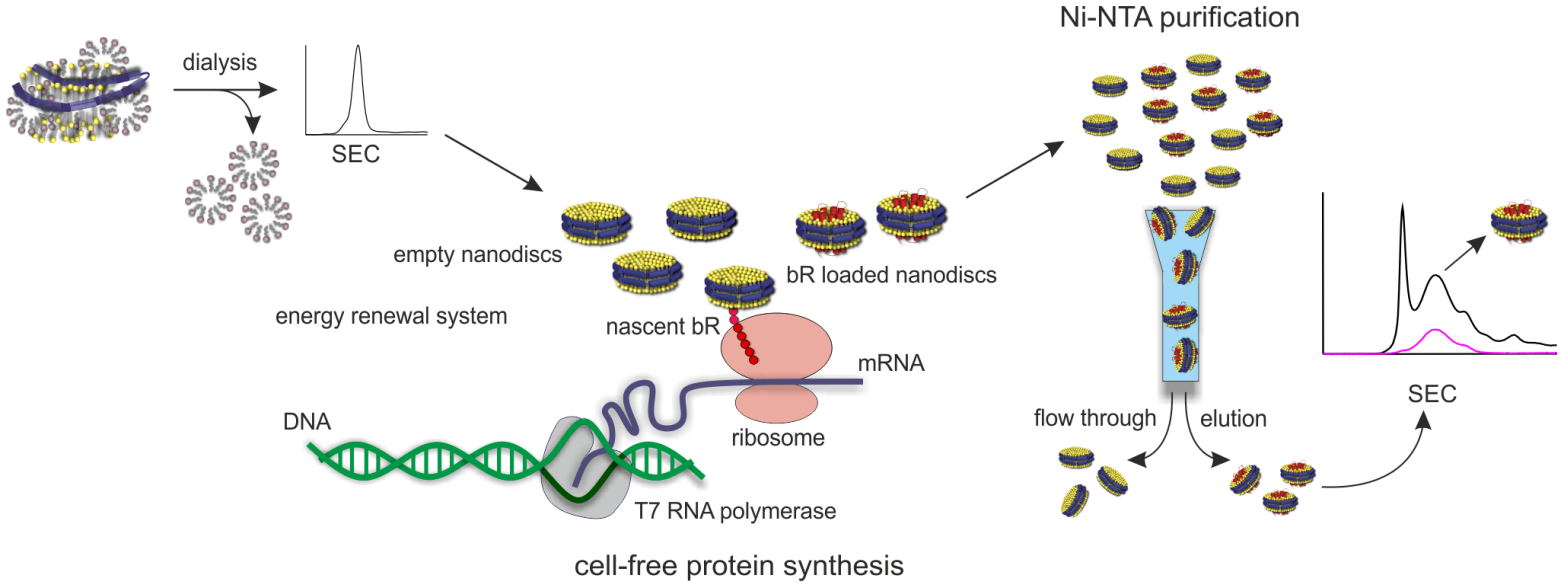
Scheme for the preparation of bR loaded nanodiscs. Empty nanodiscs were generated by incubating the lipid and MSP1 in the presence of a detergent. Detergent was removed by dialysis and nanodiscs were purified by size exclusion chromatography. The empty nanodiscs were added to the cell-free protein synthesis reaction. bR was incorporated co-translationally into nanodiscs and achieves correct folding. bR loaded nanodiscs were separated from empty nanodiscs by Ni-NTA and are further purified by size exclusion chromatography. bR was shown to be properly folded by its absorption maximum at 550 nm.

Katzen et al. [25] reported on a cell-free protein synthesis approach that leads to direct assembly of bacteriorhodopsin (bR) into nanolipoprotein particles during expression. Although the approach of creating bR loaded nanodiscs described by Bayburt et al. [23], [24] is potentially more efficient, we were interested in a more general approach allowing us the assembly of different membrane proteins into nanodiscs without making larger modifications on the protocol. An important advantage of the cell-free procedure is that detergents, which may affect the conformation of certain membrane proteins, are avoided. Thus, cell-free protein synthesis was utilized in this work to create bR loaded nanodiscs. Therefore, empty DMPC nanodiscs with MSP1 [26] as scaffold protein were added to the cell-free reaction to allow insertion of bR into the phospholipid bilayer of the nanodiscs (Fig. 1). The successful expression of bR was clearly visible by its unique purple color after addition of the cofactor retinal indicating correct folding of the protein (data not shown) [27].

**Figure 2:**
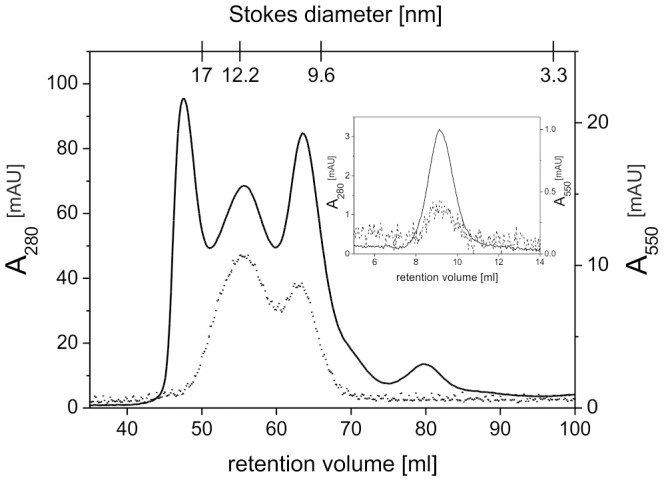
Size exclusion chromatography of bR loaded nanodiscs. Ni-NTA purified bR loaded nanodiscs were run over a HiLoad XK16/600 Superdex 200 column. Fractions containing bR loaded nanodiscs with a Stokes diameter of 12 nm were pooled and reinjected on an analytical Superdex 200 10/300GL column (inset) to verify stability of the bR nanodiscs. Solid and dotted lines show absorbance at 280 nm and 550 nm, respectively.

bR loaded nanodiscs were separated from empty nanodiscs by Ni-NTA utilizing the C-terminal His-tag of bR. Size exclusion chromatography was carried out subsequently to separate nanodisc-inserted bR from other potential bR species. The presence of bR was monitored via its absorption maximum at nearby 550 nm, whereas the overall protein was detected at 280 nm (Fig. 2). The chromatogram shows that the largest amount of functional bR elutes at a retention volume corresponding to a Stokes diameter of about 12 nm. Another peak, that shows higher absorbance at 280 nm and lower 550 nm absorbance, elutes after this peak and implicates particles with a Stokes diameter of 10 nm. According to previous work by Bayburt et al. [24], reporting that multiple copies of monomeric bR fit into one nanodisc, the chromatogram indicates the formation of different populations of bR loaded nanodiscs. Size heterogeneity in the SEC profile was also observed in approaches utilizing cell-free protein synthesis to create bR-nanolipoprotein complexes [28]. While the peak harboring the larger amount of bR probably contains nanodiscs with multiple copies of bR, the following peak however may consist of nanodiscs with only one bR molecule. Additionally, a peak coeluting with the void volume of the column does not contain any protein, but is probably due to larger structures formed by lipids [28].

**Figure 3:**
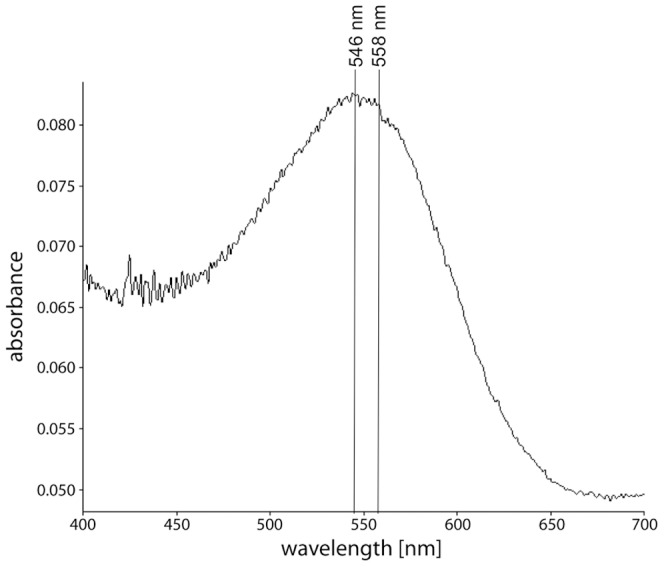
Absorption spectrum of dark-adapted bR nanodiscs. An absorption spectrum of bR nanodiscs was recorded. The absorption maximum of about 550 nm indicated correctly folded bR. The spectrum showed a maximum at 546 nm indicating bR to be predominantly in the monomeric state, whereas a maximum at 558 nm would have indicated a trimeric state.

To ensure the largest possible amount of bR for biopanning, fractions containing nanodiscs with multiple copies of bR were pooled and used as phage display target. An aliquot of those fractions was reinjected onto an analytical SEC column (Fig. 2, inset). The bR nanodiscs eluted as a single peak verifying stability and homogeneity of the sample. The visible absorption spectrum of this sample shows a maximum at 546 nm (Fig. 3). This is characteristic for dark-adapted bR monomers, while trimeric bR would show in its dark-adapted state a maximum at 558 nm [27]. Thus, even if multiple copies are inserted in the same nanodisc, they do not form a trimer. The cell-free synthesis approach of inserting bR into nanodiscs leads almost exclusively and regardless of the size of the membrane scaffold protein to monomeric bR [25], probably because incorporation of bR molecules during cell-free expression is random concerning the orientation of the protein molecules relative to each other.

### Phage selection

**Figure 4:**
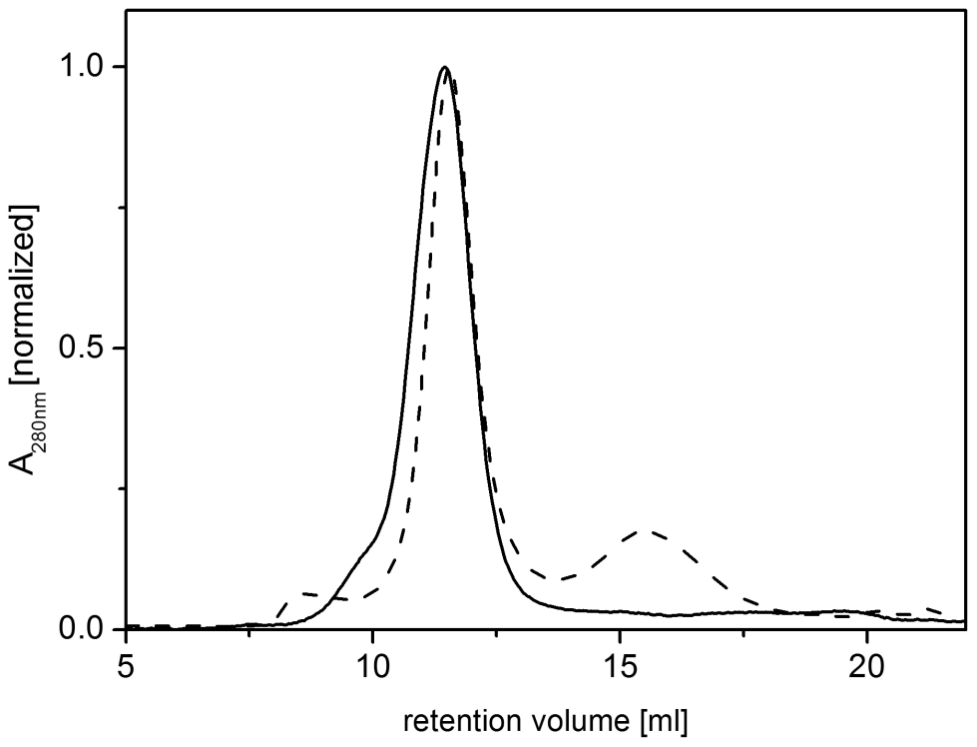
Detergent stability of nanodiscs. Nanodiscs were incubated for 3 h at room temperature in TBS+0.1% Tween 20, the washing buffer used during phage selection, and separated by size exclusion chromatography using a Superdex 200 10/300 GL column. Solid lines show absorbance at 280 nm of untreated nanodiscs, dotted lines of detergent-treated nanodiscs. The majority of the nanodiscs remained stable upon detergent treatment.

To exclude decomposition of the nanodiscs during the phage selection especially caused by the low-concentrated and mild detergent in the washing buffer, a stability test was performed beforehand. Empty nanodiscs were incubated in TBS+0.1% Tween 20 at room temperature for 3 hours and subjected to a Superdex 200 10/300 GL column. The size exclusion chromatography profile shows that there were only minor changes induced by the incubation of the nanodiscs (Fig. 4). Therefore, we considered the use of 0.1% Tween in the washing buffer as unproblematic.

**Figure 5:**
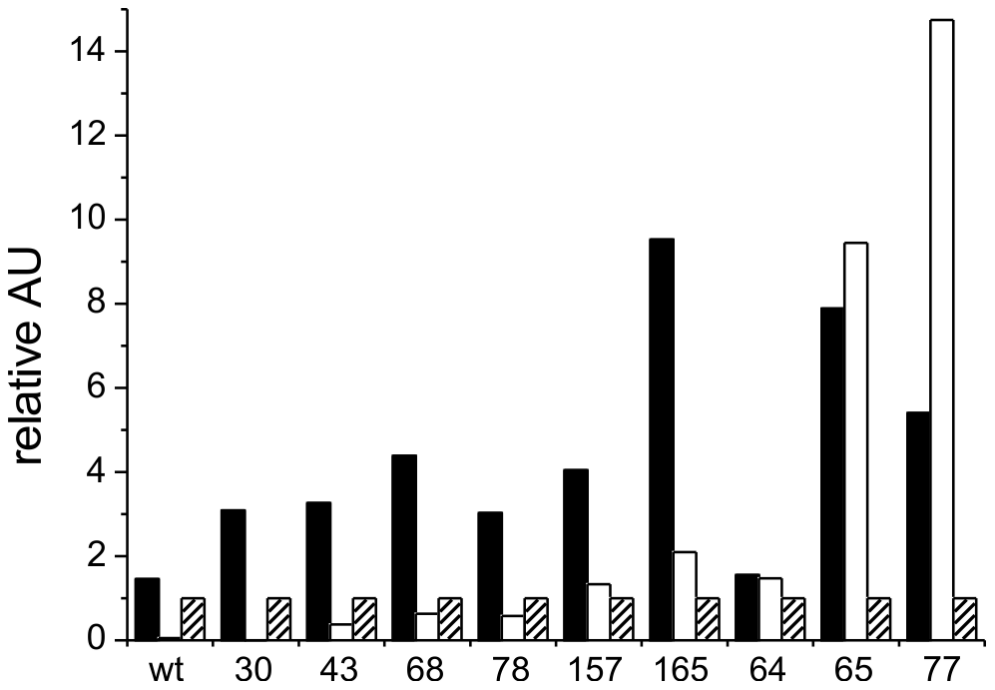
Antiphage ELISA readout values of representative phage clones selected from a 12-mer phage display peptide library. Solid bars indicate binding of the phage clone to bR loaded nanodiscs, open and hatched bars to wells of the micro titer plate that were loaded with empty nanodiscs or BSA, respectively. Readout values of the wells loaded with bR and empty nanodiscs were normalized to the well binding signal of each clone. Single determination was performed for each clone. Numbers below the bars refer to the given ID numbers of each phage clone. Wild type (wt) refers to phage displaying no peptide.

To select peptide ligands against parts of bR that are solvent accessible and not embedded into the membrane, bR was reconstituted into nanodiscs and used as target to be screened by the M13 phage library presenting random dodecapeptides. bR loaded nanodiscs were captured on Nunc Immobilizer Amino plates and incubated with the phage display library. Unbound phages were removed by washing and bound phages were eluted by lowering the pH. After four rounds of biopanning, single clones were randomly picked from the third and fourth round of biopanning and analyzed in an antiphage ELISA using an HRP-conjugated anti-M13 monoclonal antibody to quantify bR-bound phages. To eliminate false positive clones, binding of each phage clone to empty nanodiscs and to BSA coated wells of the micro titer plate was also tested. In total, 171 clones were analyzed. Figure 5 shows the representative results of nine of them. Clones were considered as positive, when the readout values derived from wells treated with bR nanodiscs were significantly higher than the readout values from nanodiscs and BSA treated wells. On the other hand, clones were considered as nanodiscs binding, when the readout values from nanodiscs treated wells were as high or higher than the readout values from bR nanodiscs treated wells. In total, 69 clones out of 171 were considered as positive, while 33 clones were found to bind to empty nanodiscs.

### Sequence determination of bR binding and nanodiscs binding peptides

The amino acid sequences of phage displayed peptides were deduced by sequencing the DNA of the 48 most promising bR binding phages and four phages binding to empty nanodiscs. For the bR binding phages, two of the sequences were obtained multiple times. However, the deduced peptide sequences did not show obvious sequence homologies with each other (see table 2a for representative clones and table S1 for the complete list of sequences). Four of six sequences shown in table 2a can be aligned along a glutamine residue, but except for a few residues, no striking similarities were visible. Thus, a consensus sequence was not determined. The clones 43, 68 and 165 were chosen for further analysis since they showed the strongest, bR specific readout values in the antiphage ELISA.

**Table 2:** Sequence alignment of peptides displayed on the surface of representative bR binding phage clones (a) and of nanodisc binding phage clones (b).

a		
ID number	frequency	sequence
165	1	AVFSQLPRTPHL
68	1	SITTQTAIYFP
43	1	LTSAISPQHGEY
157	2	HHQNTYANYPRH
30	7	GPLKAYILPPKA
78	1	LSSSAVTNNTSS

b		
ID number	frequency	sequence
65	1	KVWSVPQLSHQL
77	1	KVWLLETSHISL
64	2	KLWYLNPPPGSF

The sequences of the exposed peptides of the phages that bound empty nanodiscs revealed striking similarities to each other (table 2b). In particular, they were nearly identical in their first three amino acid residues. This sequence motif containing a lysine followed by a hydrophobic valine or leucine and a tryptophan was therefore considered as targeting empty nanodiscs. Thus bR binding phages selected in the single phage ELISA showing this motif were excluded from further analysis.

### Analysis of clones and identification of binding sites

To show that the selected bR binding phages were directed against the extramembranous regions of bR, the binding of these phages to the loops and the C- and N-termini of bR was analyzed by ELISA. Therefore, biotinylated peptides were synthesized consisting of all six loops, both in linear and cyclic form, and of the C- and N-terminus of bR (table 1). Cyclization of the loops was thought to mimic the epitope closer to its native state and was achieved by flanking the peptides with cysteine residues to allow formation of disulfide bonds. The peptides were captured on streptavidin coated plates and incubated with the phages selected from the phage display. Binding of the phages was detected via an HRP conjugated anti-M13 antibody. A clone binding to empty nanodiscs, clone 77, was included as control to check whether binding of the chosen bR binding phages is due to their specific, exposed peptide. In addition to the selected phage clones, a wild type M13 phage exposing no foreign peptide was analyzed in order to exclude reactivity of the phage virion with the bR peptides.

**Figure 6:**
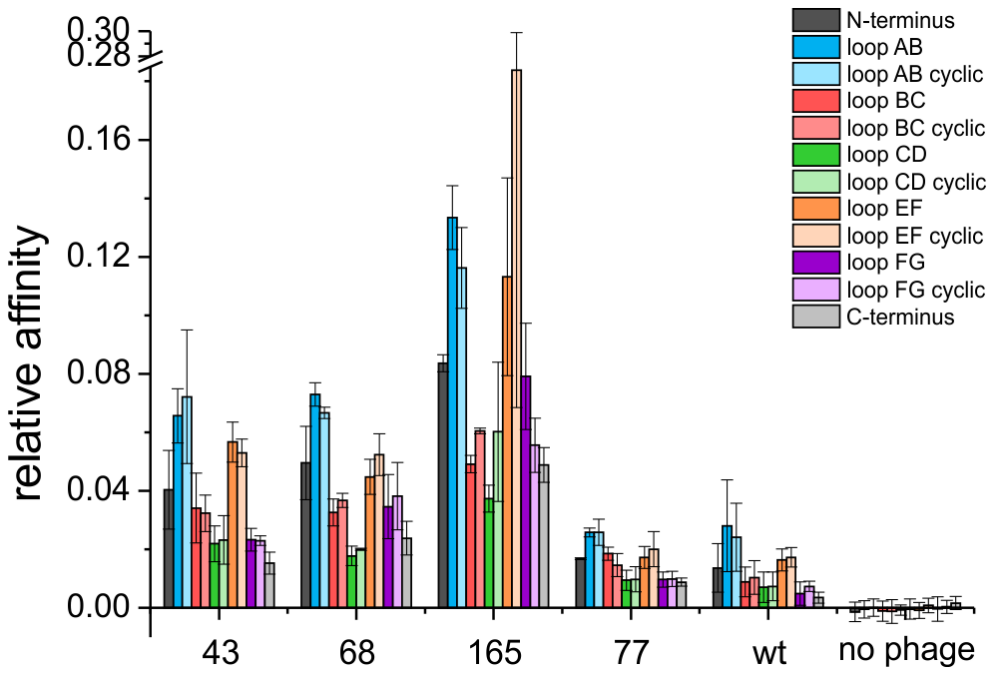
In vitro epitope mapping indicating binding of selected phage clones to extramembranous regions of bacteriorhodopsin. Biotinylated peptides representing loops and termini of bacteriorhodopsin were immobilized on streptavidin coated plates. Peptides representing loops were provided in linear and cyclic conformation. Binding of selected phages to the peptides was quantified by antiphage ELISA. Normalized mean values from three independent experiments are shown. Numbers below the bars refer to the given ID numbers of each phage clone. Wild type (wt) phages without any peptide insert served as negative control. Peptides containing loop DE of bR were not included in the experiments, since cross-reactivity with the wild type phage was observed.

Elevated binding levels of the tested clones, 43, 68 and 165, were found for loop AB, both for the cyclic and the linear form. In addition, the clones 43 and 165 showed also high binding signals to the linear and cyclic peptide of loop EF (Fig. 6). Thus, the selected bR binding phages are indeed targeting regions that protrude from the membrane. Moreover, the results implicate that loop AB and to some extent loop EF, which are both located on the intracellular side of the protein, seem to form a possible epitope for the selected bR binding clones.

In general, no significant difference between the binding levels of the linear and the cyclic forms of the loop peptides could be observed. Hence, cyclisation of the peptides seems to have no significant influence on the binding behavior. In comparison to the bR binding phages, the wild type phage and the nanodisc binding phage clone 77 showed only low binding levels to the bR peptides indicating that the displayed peptides of the selected bR binding phages are necessary for the binding to loops AB and EF. The peptide representing loop DE, however, revealed very high signals upon incubation with all clones, even with the wild type phage, suggesting cross-reactivity of this peptide with a phage surface protein (data not shown). Cross-reactivity of the anti-phage antibody with the loop peptides could be excluded, since no signals were detected in absence of phages.

## Discussion

Phage display selection directed against integral membrane proteins is a major challenge and was achieved in the past only by intricate selection strategies (for a review see [29]). In this work, we showed that integral membrane proteins can be prepared and used as targets for phage selection by using nanodiscs as the ideal membrane environment. We used bR as a model for a polytopic membrane protein. With its seven-transmembrane helices, bR requires a membrane environment for proper folding and epitope formation.

Nanodiscs were used to mimic the membrane environment of bR and to keep it into a soluble form without the need of detergents, which are typically used to solubilize membrane proteins. The assembly of bR into the nanodiscs was achieved successfully using cell-free expression and bR functionality was confirmed by its absorption maximum near 550 nm. Nanodiscs were obtained containing multiple copies of monomeric bR. Although the native form of bR is a trimer, the photocycle of bR monomers is qualitatively the same [30]. Assembled into nanodiscs, bR behaves like a soluble protein and displays native epitope formation.

The screening of a 12-mer phage display library against bR containing nanodiscs, led to the selection of 69 potentially positive phage clones binding specifically to bR. Peptides resembling each of the extramembranous regions were used to elucidate the binding sites of three bR binding phages. The analysis revealed that the phages bind to intracellular, extramembranous loops of bR. The binding was independent on whether the loop peptides were provided in linear or in cyclic form.

**Figure 7:**
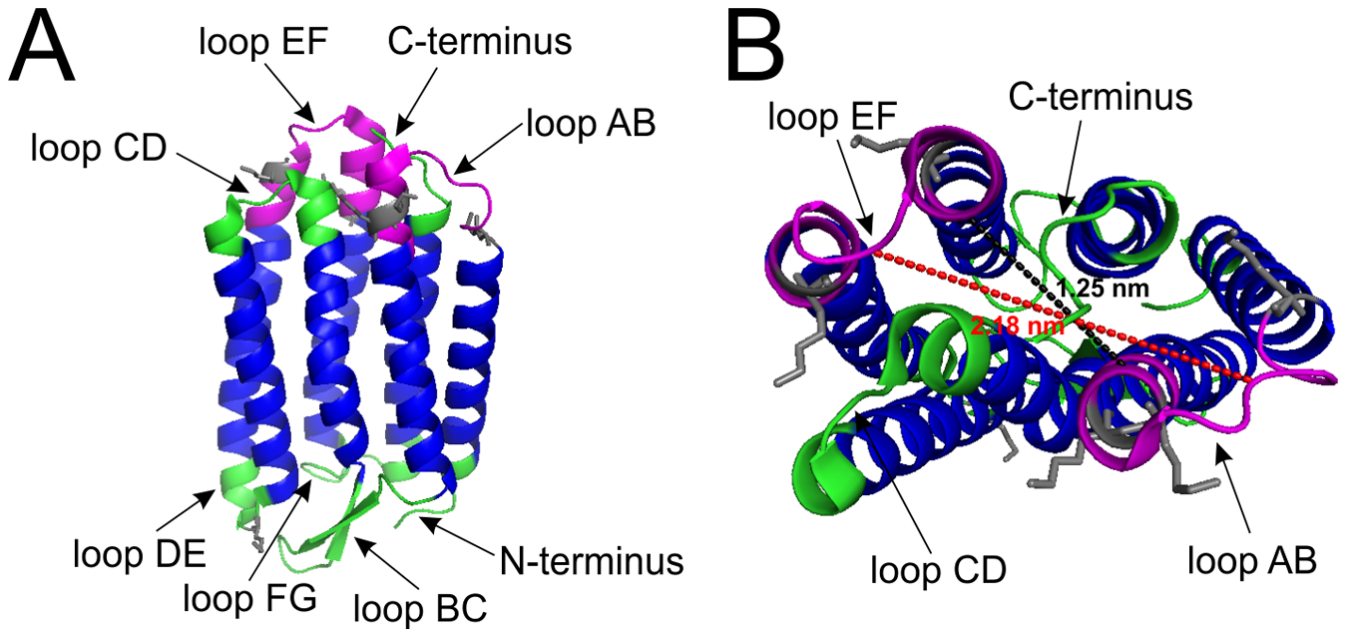
Crystal structure of bacteriorhodopsin (PDB accession number 1IW6). **A** shows a side view of bR and **B** a view from above. Transmembrane regions are shown in blue and extramembranous regions are shown in green and purple. Putative binding sites for the phage clones 43, 68 and 165 are highlighted in purple. Dotted lines indicate distances between residues of the loops AB and EF: black dotted line between the C^α^ of the residues A38 and V167 (1.25 nm) and red dotted line between the C^α^ of the residues V34 and M163 (2.18 nm). Lysine residues including their side chains are marked in grey. Structure visualization of bR was done with pymol.

The selected clones interact with the loops AB and EF of bR, but not with loop CD, which is also located on the intracellular side. Loops AB and EF are larger than loop CD, which might hardly be solvent accessible (Fig. 7). Furthermore, the crystal structure shows that the loops AB and EF are higher and overtop loop CD. Thus, one may speculate, whether interaction with potential ligands on the intracellular side might take place preferentially at loops AB and EF. Based on the crystal structure, the distance between residues of both loops is between 1.3 nm and 2.2 nm (Fig. 7). Since 12-mer peptides in extended conformation have a length of about 4.2 nm, even in their not fully extended conformation, the peptides are able to cover both loops at the same time. The C-terminus, which is relatively long, however, was not involved in one of the assayed interactions. NMR experiments [31] have shown that the C-terminus of bR starting at Pro236 is mobile, while the first part of the C-terminus is constrained to the membrane. Therefore, the C-terminus might not be suitable to form an epitope for interactions with water soluble ligands. Since the identified epitope consists of at least two linear stretches of amino acid sequences in the target protein, as shown by the binding activities to loop AB and EF, we conclude that these loops form a non-linear, discontinuous epitope for the binding of ligands, e.g. the selected phages.

We can only speculate, why none of the selected clones bind to the extracellular surface. bR has evolved in *Halobacterium salinarum* as a light-driven proton pump and although being localized on the surface of the cell, has no function as a receptor. It may well be that during evolution the extracellular side was selected against having discrete binding pockets. It is unlikely that the extracellular surface was inaccessible during biopanning. We used covalent coupling via lysine residues to immobilize the bR nanodiscs on the plate. Five lysine residues are located at the intracellular side in loops AB and EF and only one lysine residue is located at the extracellular side in loop DE. The side chains of the lysine residues of both sides are protruding into the solvent and seem to be well accessible. From the structure it is not clear whether one of these lysines is favored for immobilization on the plate (Fig. 7). The most lysines by far (18) are present in the scaffold protein of the nanodiscs. Since it is possible to immobilize empty nanodiscs on amino plates, we can assume that at least some of these lysines are accessible for immobilization, too. Therefore, it is most likely that immobilization of the bR nanodiscs took place randomly.

Many attempts were made to target membrane proteins by phage display selections. Our strategy that uses membrane proteins embedded into nanodiscs allows a simple and universal preparation of the target at a close to physiological state. The cell-free expression of the membrane protein and its simultaneous incorporation into preformed, empty nanodiscs allows a simple straightforward preparation of the target. Since only small amounts of protein (500 µg in total) are necessary for panning and further analysis of about 170 selected phages, the yield obtained from 1 ml of cell-free protein synthesis is sufficient for the complete phage display selection process. It would have been easy to consume significantly less protein. Additionally, the risk of denaturating the target protein during preparation by detergents is circumvented. In principle, the phage displayed peptide library can be replaced straightforwardly by phage displayed antibody or Fab fragment libraries.

We have shown that membrane proteins, when prepared in nanodiscs, can be used straightforwardly as targets in phage display selection and that it is possible to select clones binding to non-linear epitopes. Since membrane proteins represent the largest class of drug targets, this approach may be of great value in drug design and development.

## Supporting Information

Table S1 **Multiple sequence alignment of all peptides sequences exposed by bR binding clones.**
(DOCX)
