# Supplementary material for: Nanodiscs Allow Phage Display Selection for Ligands to Non-Linear Epitopes on Membrane Proteins
Source: PLoS One. 2013 Sep 9;8(9):e72272. doi: 10.1371/journal.pone.0072272 (PMC3767683; doi:10.1371/journal.pone.0072272)
Supplement: Table S1 — Multiple sequence alignment of all peptides sequences exposed by bR binding clones. (DOCX) [file pone.0072272.s001.docx]

| ID number | frequency | sequence |
| --- | --- | --- |
| 191 | 1 | NQYMSNGLVWAL |
| 197 | 1 | GDWPAGQMARSA |
| 217 | 1 | SPWSPPFWNDDM |
| 48 | 1 | QGPQTDSAPPRF |
| 212 | 1 | GFNTNPPSFPRP |
| 30 | 7 | GPLKAYILPPKA |
| 143 | 1 | AAHQPPAQSDFL |
| 209 | 1 | WSAPGLSSSSAP |
| 78 | 1 | LSSSAVTNNTSS |
| 186 | 1 | DSPTVAHNTSPT |
| 137 | 1 | HETVQHNKGWMI |
| 139 | 1 | IEHNGKAWRIPQ |
| 142 | 1 | EVAAFSMPGRFS |
| 201 | 1 | YVEPQEQSMPYL |
| 198 | 1 | NIQLELNPRHLI |
| 156 | 1 | SNSTREFNPNMF |
| 157 | 2 | HHQNTYANYPRH |
| 42 | 1 | WPHHHSRHNHNH |
| 205 | 1 | STHGWMNDRHHP |
| 17 | 1 | KSITSNDGFNTL |
| 68 | 1 | GSITTQTAIYFP |
| 43 | 1 | LTSAISPQHGEY |
| 136 | 1 | SHSFHTQERTTH |
| 165 | 1 | AVFSQLPRTPHL |
| 150 | 1 | SLPEAPIRQYQG |
| 196 | 1 | HVTMSWPQTAQN |
| 151 | 1 | SNAGGLMSRTWE |
| 213 | 1 | KIMPDSWAIKPW |
| 215 | 1 | NVMIDKHNVNGS |
| 208 | 1 | DSYMHAYSWRTK |
| 195 | 1 | SFQNSTLHGPVY |
| 57 | 1 | HIGHDNHLYPNR |
| 41 | 1 | NARVLHTGNESL |
| 129 | 1 | DRAMTPIYNPYI |
| 73 | 1 | TYTDNGYFKRST |
| 153 | 1 | TATIKSEAKSPS |
| 12 | 1 | YVEQVSTGKARS |
| 21 | 1 | RTRMRSINSPNL |
| 172 | 1 | SYYGKTDTADLT |
| 66 | 1 | VVDIRSQFANQQ |
